# Vision-related parameters that affect stereopsis in patients with macular hole

**DOI:** 10.1038/s41598-020-59844-0

**Published:** 2020-02-18

**Authors:** Fumiki Okamoto, Shohei Morikawa, Yuki Moriya, Yoshimi Sugiura, Tomoya Murakami, Mizuki Tomioka, Takahiro Hiraoka, Tetsuro Oshika

**Affiliations:** Department of Ophthalmology, Faculty of Medicine, University of Tsukuba, Ibaraki, Japan

**Keywords:** Retinal diseases, Vision disorders

## Abstract

This study evaluated stereopsis and other visual functions in patients with idiopathic macular hole (MH), and sought to identify vision-related parameters that affect stereopsis. In this prospective, consecutive, comparative study, 39 eyes of 39 patients with unilateral idiopathic MH were included. At baseline and at 6 months after MH surgery, we evaluated stereopsis, with the Titmus stereo test (TST) and TNO stereotest (TNO), best-corrected visual acuity (BCVA), letter contrast sensitivity, severity of metamorphopsia, as assessed using M-CHARTS, and extent of aniseikonia, by the new aniseikonia test. Preoperative stereopsis (log) in patients with MH were 2.72 ± 0.53 (range 1.9–4.1) in the TST and 2.82 ± 0.65 (range 1.8–3.9) in the TNO. Preoperative TST was significantly correlated with letter contrast sensitivity (p < 0.05), but not with the other visual functions. TNO showed significant correlation with letter contrast sensitivity (p < 0.05) and aniseikonia (p < 0.005). Preoperative TNO was associated with aniseikonia by multivariate analysis (p < 0.005). MH surgery significantly improved stereopsis, BCVA, letter contrast sensitivity, metamorphopsia, and aniseikonia. Postoperative TST and TNO was significantly associated with BCVA by multivariate analysis. Deterioration of stereopsis in MH patients is associated with contrast sensitivity and the degree of aniseikonia.

## Introduction

Macular hole (MH) causes not only blurred vision, but also various other visual impairments, such as metamorphopsia^1–7^, aniseikonia^8,9^, and stereopsis^10–12^. Visual acuity improves in many patients after successful MH surgery^13,14^, whereas some patients still complain of disturbances in visual function after surgery.

Stereopsis is the ability to perceive depth of field based on the disparity between the images formed by the two eyes—it is the most advanced visual function. Wheatstone invented the stereoscope and discovered that, if areas on the retina of both eyes that are separated horizontally were stimulated simultaneously, stereopsis could occur^15^. Visual acuity is the most commonly considered vision-related parameter that affects stereopsis. Stereopsis worsens even when visual impairment is present in only one eye^16–20^. In addition, some other factors are known to affect stereopsis, such as aniseikonia^21–23^, pupil size^23,24^, eye dominance^21,25^, and accommodation^20,26^.

Several studies have investigated stereopsis in retinal disorders, including MH^10–12^ and epiretinal membrane (ERM)^12,27^, and after retinal detachment (RD) surgery^28^, all of which impaired stereopsis. However, the details of vision-related parameters that cause those disturbances of stereopsis in retinal disorders remain unknown.

The purpose of the present study was to evaluate stereopsis and other visual function parameters, including visual acuity, contrast sensitivity, metamorphopsia, and aniseikonia in patients with unilateral MH, and to identify vision-related parameters that affect stereopsis.

## Methods

We conducted this prospective, consecutive, comparative study in accordance with the Declaration of Helsinki and received approval from the institutional review committee of the University of Tsukuba Hospital. Prior to inclusion in the study, all patients provided informed consent after the nature of the study was explained.

We investigated patients with unilateral, idiopathic MH, who were followed up for 6 months after surgical treatment at the University of Tsukuba Hospital. Exclusion criteria included eyes with traumatic MH, MH secondary to proliferative diabetic retinopathy or uveitis, ophthalmic disorders except mild refractive errors and mild cataract, any systemic disease that influenced ocular motility, and vitreoretinal disorders except MH. Eyes with more than 2.0 diopters of anisometropia were also excluded from the study. We included 39 eyes of 39 patients with unilateral, idiopathic MH; these included 17 male and 22 female patients, with an average age of 66.1 ± 6.1 years (means ± SD).

### Visual examinations

Patients were examined for stereopsis, best-corrected visual acuity (BCVA), letter contrast sensitivity, severity of metamorphopsia and extent of aniseikonia before surgery and 6 months after surgery.

Stereopsis was measured with the Titmus stereo test (TST) and TNO stereotest (TNO) at the standard viewing distance of 40 cm with appropriate spectacle correction. To ensure that patients were not using monocular clues in TST, responses were checked by inverting the stereotarget and asking the patient whether the target appeared in front of or behind the page^27^. The results of the TST and TNO were expressed as “seconds of arc.” We converted these values to logarithms for statistical evaluation^27^.

BCVA was measured with the Landolt Chart and expressed as a logarithm of the minimum angle of resolution (logMAR).

We examined letter contrast sensitivity by using the CSV-1000LV chart (Vector Vision, Greenville, OH). This chart consists of 24 letters, of which three letters having the same contrast are shown. Each subsequent triplet has reduced contrast. If no letter is detected, the participant receives a score of 0, while the highest score is 24. We performed the test monocularly with the best spectacle correction in an undilated state at a distance of 2.5 m.

Severity of metamorphopsia was assessed using M-CHARTS (Inami Co., Tokyo, Japan). The M-CHARTS consists of 19 double dotted lines, and the dot spacing is at a visual angle of 0.2 to 2.0 degrees. When a continuous line is substituted with a dotted line and the dot interval is changed from fine to coarse, the line distortion reduces with increasing dot intervals until the line appears to be continous^29,30^. If the patient recognized a straight line (0 degrees) as irregular or curved, we continued with the M-CHARTS, where the intervals of the dotted line gradually changed from fine to coarse on subsequent pages. When the patient recognized a dotted line as straight, the visual angle separating the dots was determined to represent the patient’s metamorphopsia score. We performed the test monocularly with the best spectacle correction in an undilated state at a distance of 30 cm. Both vertical and horizontal meridians were assessed, and their mean values were used for data analyses.

The degree of aniseikonia was assessed using the new aniseikonia test (NAT) (Handaya, Tokyo, Japan). The NAT is a simple method for quantifying the degree of aniseikonia^31^. The test consisted of matched pairs of red/green semicircles with a target size of 4 cm and allowed for measurement of the degree of aniseikonia from 1% to 24%. Two semicircles (red and green) with different sizes in each pair were arranged in a series, with the difference varying in increments of 1%. The subject, wearing red/green spectacles, viewed the plates to allow for the right eye to see one of the semicircles and for the left eye to see the other semicircle in each pair. The subjects indicated the pair in which the 2 semicircles seem to be of equal size. The actual size difference in the semicircles in the pair represents the degree of aniseikonia in the subject. The subjects were examined at a distance of 40 cm, in both vertical and horizontal meridians, and their mean values were used for data analyses. Aniseikonia of 2% or greater was considered macropsia and aniseikonia of −2% or less as micropsia.

The retinal microstructure was measured with spectral-domain OCT (Cirrus high-definition OCT; Carl Zeiss, Dublin, CA, USA). We used 5-line raster scans for each eye using an analytical software package (Cirrus analysis software, version 3.0; Carl Zeiss) with a signal strength of more than 7/10. We quantified the following parameters before and after surgery based on OCT images: minimum diameters of MH, base diameters of MH and external limiting membrane (ELM). Based on the images of the 5-line raster scans, we quantified the parameters with an image processing software (ImageJ, National Institutes of Health, Bethesda, MD). The defect length of each line was determined by agreement between two blinded, well-trained observers (Y.S. and Y.M.), and the mean value of the length of each line was used for further analysis.

### Macular hole repair surgery

All surgeries were performed by two surgeons (F.O., Y.S.), using standard pars plana vitrectomy with 25 gauge probes (Alcon Constellation vision system) under sub-Tenon local anaesthesia. When a clinically significant cataract was observed, it was simultaneously operated. After inducing posterior vitreous detachment and performing core vitrectomy, we injected 0.2 ml of 0.025% brilliant blue G solution gently over the macula for 15 seconds and washed it out with an irrigation solution. Internal limiting membrane (ILM) peeling and fluid/air exchange were performed in all cases. For the next 1–3 days, the patients were instructed to maintain a face-down position^8^.

### Statistical analysis

The mean scores were compared, and standard deviations were calculated for each vision-related parameter. The Wilcoxon signed-rank test was used to compare differences in visual functions before and after surgery in MH patients. Associations between stereopsis and the other parameters of visual function, including BCVA, letter contrast sensitivity, metamorphopsia, and aniseikonia were examined by the Spearman rank correlation test. Multivariate analysis with stepwise regression was performed to investigate the relationship between stereopsis and other vision-related parameters. All tests of associations were considered statistically significant if p was < 0.05. Analyses were performed using StatView (version 5.0, SAS Inc., Cary, NC).

## Results

### Clinical features and visual function following MH surgery

Table 1 shows the clinical features and visual functions in patients with MH before and after repair surgery. Preoperative BCVA was 0.74 ± 0.32 (range 0.15–1.40), and postoperative BCVA was 0.27 ± 0.28 (range −0.08–1.0). Vitrectomy significantly improved BCVA (p < 0.0001), stereopsis in the TST (p < 0.0001) and TNO (p < 0.0001), and letter contrast sensitivity (p < 0.0001). Postoperative severity of metamorphopsia and the degree of aniseikonia showed significant reduction compared to preoperative values (p < 0.0001 and p = 0.014, respectively). Preoperatively, 30 eyes were phakic and 9 eyes were pseudophakic with posterior chamber intraocular lenses. All phakic eyes were performed with cataract surgery combined with vitrectomy.

**Table 1:** Clinical features and visual functions following macular hole (MH) surgery.

	Before surgery	6 months after surgery	P values
Age (years)	66.1 ± 6.1	—	—
Sex (men/women)	17/22	—	—
Stage of MH (2/3/4)	(8/21/10)	—	—
Best-corrected visual acuity (logMAR)	0.74 ± 0.32	0.27 ± 0.28	<0.0001*
Titmus Stereo Test (TST) (log)	2.72 ± 0.53	2.19 ± 0.35	<0.0001*
TNO stereotest (log)	2.82 ± 0.65	2.32 ± 0.41	<0.0001*
Letter contrast sensitivity (letters)	16.3 ± 4.7	21.3 ± 2.1	<0.0001*
Severity of metamorphopsia (°)	0.89 ± 0.57	0.48 ± 0.33	<0.0001*
Degree of aniseikonia (%)	3.51 ± 3.57	1.44 ± 1.54	0.014*
Minimum diameters of MH (µm)	394 ± 180	—	—
Base diameter of MH (µm)	811 ± 251	—	—
Defect length of external limiting membrane (ELM) (µm)	807 ± 327	1.2 ± 7.1	<0.0001*

Values are presented as the mean ± standard deviation.

LogMAR = logarithm of the minimum angle of resolution.

*Statistically significant compared with baseline.

### Relationship between stereopsis and OCT parameters before surgery

The preoperative TST values showed significant correlation with the minimum diameters of MH (p < 0.05), whereas other variables including the base diameter of MH and the ELM defect length were not relevant. TNO values were significantly associated with the base diameter of MH (p < 0.05), whereas other variables were not relevant. Postoperative TNO values showed significant association with preoperative ELM defect length (p < 0.05). There was no significant association between MH stage and stereopsis (Bonferroni test).

### Relationship between stereopsis and other visual functions before surgery

Preoperative stereopsis in TST had a significant correlation with preoperative letter contrast sensitivity, but not with other visual functions. Preoperative stereopsis in the TNO was significantly associated with preoperative letter contrast sensitivity and the degree of aniseikonia (p < 0.05 and p < 0.0005, respectively) (Table 2). In stepwise multiple regression analysis, preoperative TNO showed a significant association with preoperative aniseikonia (p < 0.05).

**Table 2:** Correlation between preoperative stereopsis and the other visual functions in patients with macular hole (MH).

Preoperative parameters	Preoperative TST		Preoperative TNO	
	r	p	r	p
Best-corrected visual acuity	0.23	0.21	0.17	0.40
Letter contrast sensitivity	−0.32	0.03*	−0.30	0.03*
Severity of metamorphopsia	−0.05	0.66	−0.147	0.26
Degree of aniseikonia	0.29	0.09	0.62	0.0002*

*Significant correlation between the parameters (Spearman rank correlation test).

TST = Titmus Stereo Test, TNO = TNO stereotest.

### Relationship between stereopsis and other visual functions after surgery

At 6 months postoperatively, stereopsis in the TST correlated significantly with BCVA and the degree of aniseikonia (p < 0.05 and p < 0.01, respectively) (Table 3). Postoperative TST was associated with postoperative BCVA by multivariate analysis (p < 0.05). Postoperative stereopsis in TNO was significantly associated with postoperative BCVA (p < 0.05).

**Table 3:** Correlation between postoperative stereopsis and the other visual functions in patients with macular hole (MH).

Postoperative parameters	Postoperative TST		Postoperative TNO	
	R	p	R	p
Best-corrected visual acuity	0.39	0.03*	0.41	0.04*
Letter contrast sensitivity	−0.27	0.07	−0.10	0.07
Severity of metamorphopsia	0.19	0.31	0.16	0.56
Degree of aniseikonia	0.52	0.006*	0.40	0.07

*Significant correlation between the parameters (Spearman rank correlation test).

TST = Titmus Stereo Test, TNO = TNO stereotest.

### Preoperative visual function factors affecting postoperative stereopsis

Postoperative stereopsis in the TST and TNO showed no association with any visual functions, including postoperative BCVA, letter contrast sensitivity, severity of metamorphopsia, and the degree of aniseikonia.

## Discussion

Stereopsis in both the TST and TNO was compromised in patients with MH, and MH surgery improved visual acuity, contrast sensitivity, metamorphopsia, and aniseikonia, as well as stereopsis in this study.

Previous studies have reported the impairment of stereopsis in patients with unilateral ERM and RD^27,28^. Stereopsis was affected in MH patients in a way similar to that in patients with other unilateral retinal diseases^12^. Surgery for ERM and RD also improved stereopsis^27,28^, although not to a normal level. Postoperative stereopsis (log) in patients with MH in this study were 2.19 in the TST and 2.32 in the TNO. In comparison, postoperative stereopsis in ERM patients were 2.19 in the TST and 2.63 in the TNO^27^, and stereopsis after surgery for RD were 2.43 in the TST and 2.94 in the TNO^28^. Thus, even after treatment, patients with MH had stereopsis that was as poor as that in patients with other retinal diseases.

The preoperative TST score was significantly associated with letter contrast sensitivity, but not with other vision-related parameters in our study. Stereopsis is affected by visual acuity^16–20^. If the visual acuity of one eye of a healthy subject who can discriminate circle 9 in the TST was impaired to 20/200 by cycloplegic condition with a convex lens, only TST circle 3 can be discriminated. In addition, the degree of visual impairment and stereopsis have been shown to be significantly correlated^19^. In the present study, stereopsis was related to contrast sensitivity, but not to visual acuity. Visual acuity and contrast sensitivity are both indicators of form sense, but both can be understood as parameters of independent visual functions. Tranos *et al*. reported that MH surgery improved visual acuity but did not affect contrast sensitivity^32^. Monestam *et al*. found that contrast sensitivity in patients with MH was not associated with visual acuity^33^. The index of letter contrast sensitivity used in our study was similar to the Pelli–Robson chart, and the spatial frequency was constant, with only changes in contrast. Thus, stereopsis may be disturbed even if the ability to distinguish contrast is impaired in only one eye.

The preoperative TNO score was significantly associated with the degree of aniseikonia according to multivariate analysis. Previous reports found that the degree of aniseikonia affected stereopsis^21–23^. Brooks *et al*. reported that, even if experimental anisometropia of 1 diopter (about 1% of aniseikonia) was created in normal subjects, stereopsis broke down^34^. The mean degree of aniseikonia of MH patients was 3.51% in the present study; therefore, aniseikonia in MH patients may contribute to the impaired stereopsis.

The postoperative TST and TNO scores revealed significant correlation with visual acuity, but not with contrast sensitivity and aniseikonia in MH patients. As the mean degree of aniseikonia was improved from 3.51% to 1.44% by surgery, the postoperative awareness of aniseikonia in patients with MH was considered to have essentially disappeared. Therefore, under good visual function, stereopsis and visual acuity may be related, as indicated in previous reports^16–20^.

Our study had several limitations, particularly its small sample size and short follow-up duration. Visual acuity and stereopsis in MH patients may improve over a longer follow-up period. Although some other factors are known to affect stereopsis, such as eye dominance^21,25^, pupil size^23,24^, and accommodation^20,26^, we did not evaluate these factors in this study. However, the influence of pupil size and eye dominance on stereopsis is slight, and it is unlikely that our results will change. Even if the pupil size was changed from 1 mm to 6 mm, the change in TST score was 0.18 in a previous report^23^, while, if the dominant eye changed, the TST score changed by 0.2^21^. Future studies that include a larger sample size and longer follow-up duration will further improve our understanding of stereopsis and other visual functions in patients with MH. The worst preoperative BCVA was 1.40 in the subjects of this study, and this visual acuity corresponds to a visual angle of 25°. At the standard examination distance, the TST circles subtend a visual angle of 0.7°and 1 set of 4 circles approximately 2.5°. TNO stereotest subtends a visual angle of 8.5°. Thus, patients with poor vision may not be able to measure stereopsis accurately. Development of a stereopsis test that can measure even patients with poor visual acuity is desired.
